# Tailoring Acidity and Structure of ZSM‑5 and NaY Zeolites via Ion Exchange and Nb_2_O_5_ Impregnation for Enhanced Hydrocracking of Crude Soybean Oil

**DOI:** 10.1021/acsomega.6c08841

**Published:** 2026-10-09

**Authors:** Taysa S. Braniz, Ana C. R. Aranha, Thaisa F. Coslop, Marcos de Souza, Mara H. N. O. Scaliante

**Affiliations:** † Postgraduate Program in Chemical Engineering, Department of Chemical Engineering, State University of Maringá, Av. Colombo 5790, Bloco D90, Maringá, Paraná CEP 87020-900, Brazil; ‡ Department of Chemical Engineering, State University of Maringá, Av. Colombo 5790, Bloco D90, Maringá, Paraná CEP 87020-900, Brazil

## Abstract

The catalytic hydrocracking of crude soybean oil was investigated using ZSM-5 and NaY zeolites modified by ammonium ion exchange and wet impregnation with 5 wt % niobium pentoxide (Nb_2_O_5_). The aim was to correlate changes in acidity, structural properties, and product selectivity for biofuel production. Catalysts were characterized by XRD, FTIR, SEM, N_2_ physisorption, NH_3_-TPD, and atomic absorption spectroscopy. Ion exchange significantly increased total acidity, particularly for HY (241% increase) and HZSM-5 (59% increase), while Nb_2_O_5_ impregnation modified the acidity profiles and influenced the distribution of acid sites without affecting the zeolites’ framework stability. The observed changes in acidity following Nb_2_O_5_ impregnation were not uniform, suggesting a redistribution of acid site strength rather than simple neutralization. Hydrocracking reactions were carried out at 450 °C under hydrogen flow in a fixed-bed reactor. ZSM-5-based catalysts exhibited higher selectivity toward gaseous hydrocarbons (C_1_–C_5_), whereas NaY-based catalysts favored light liquid hydrocarbons (C_6_–C_8_), reflecting their weaker acid sites and larger pores. Proton-exchanged HZSM-5 enhanced gasoline-range product formation and improved deoxygenation efficiency. Niobium-impregnated catalysts promoted aromatization and shifted the deoxygenation pathway from hydrodeoxygenation toward decarboxylation over time. The results demonstrate that acidity tuning through ion exchange and Nb_2_O_5_ addition effectively controls cracking pathways and hydrocarbon distribution, offering a viable strategy for biofuel production from vegetable oils.

## Graphical abstract

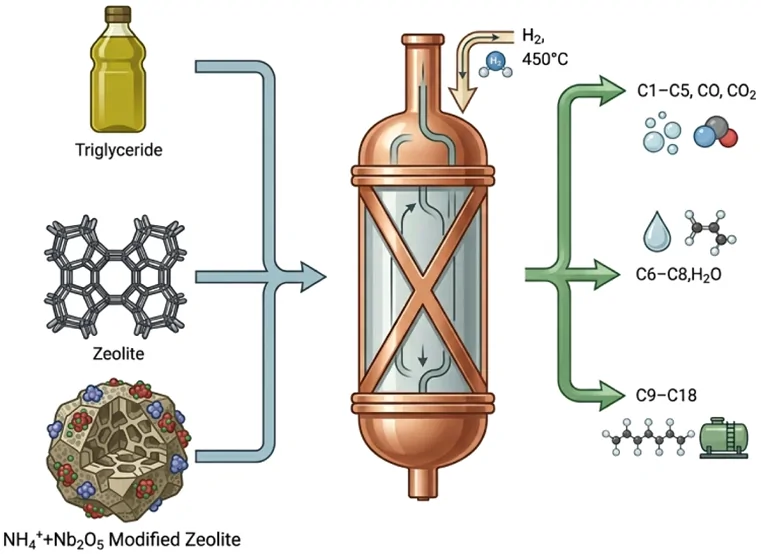

## 1 Introduction

The increasing global demand for energy, together with mounting pressure to mitigate environmental degradation, has intensified research aimed at developing alternative fuels derived from renewable resources as well as from industrial and domestic waste materials.^1–3^ The continued unsustainable consumption of fossil fuels has significantly contributed to the intensification of the greenhouse effect, leading to alterations in global climate patterns, including changes in rainfall distribution, rising sea levels, and a higher frequency of extreme weather events.^4,5^

In response to these challenges, considerable international efforts have been directed toward identifying viable renewable energy solutions. Among these, biofuels have emerged as a promising substitute or complement to petroleum-derived fuels. Brazil, in particular, presents a favorable scenario for biomass-based energy due to its high solar incidence and extensive agricultural diversity, which supports a wide range of feedstock options.^6–8^ Currently, soybean stands as the primary source of vegetable oil for biofuel production. Although these oils present strong potential as renewable feedstocks, they require chemical upgrading prior to their use in combustion engines.^9,10^

Several conversion pathways are employed to transform vegetable oils into biofuels, including esterification/transesterification, pyrolysis, and catalytic cracking.^11,12^ Transesterification involves the reaction of a short-chain alcohol with triglycerides to form fatty acid alkyl esters and glycerol; however, its industrial viability is limited by high operational costs and challenges related to byproduct purification.^13,14^ Pyrolysis allows thermal decomposition of oils at elevated temperatures and pressures but often leads to excessive cracking, producing large quantities of light gases and resulting in low overall biofuel yield.^15,16^ Catalytic cracking, on the other hand, is an economically attractive method capable of producing hydrocarbon fractions such as gasoline, kerosene, and diesel that are fully compatible with existing fuel infrastructure. During catalytic cracking, oxygen is removed primarily as H_2_O, CO, and CO_2_ through deoxygenation pathways.^17,18^

Zeolites are widely utilized as catalysts in cracking processes due to their well-defined microporous structures, high acidity, shape selectivity, and superior catalytic activity compared to amorphous silica–alumina materials.^19,20^ ZSM-5, in particular, is extensively applied in cracking, aromatization, isomerization, and alkylation reactions owing to its medium-pore MFI topology, structural selectivity, ion-exchange capacity, and well-distributed Brønsted acid sites.^21,22^ Nevertheless, zeolite Y remains the most widely employed zeolite in petroleum refining, especially in fluid catalytic cracking (FCC), due to its high surface area, strong acidity, and large-pore FAU framework, which facilitates the conversion of bulky hydrocarbon molecules.^23,24^

Metal oxides are also recognized as efficient catalytic materials due to attributes such as high surface area, tunable redox properties, and the presence of acidic and/or basic active sites.^25,26^ Their catalytic performance is closely linked to the metal–oxygen coordination environment, surface defects, and the ability of their unsaturated metal centers to adsorb and activate reactants via hydrogenation, dehydrogenation, and deoxygenation reactions.^27,28^ These characteristics render metal oxides suitable for modifying zeolites in order to enhance acidity, adjust hydrophobicity, or introduce additional active sites. Niobium pentoxide (Nb_2_O_5_), in particular, is known for its strong acidity, redox versatility, and capacity to improve the catalytic activity and selectivity of zeolitic materials when used as a modifying phase. Unlike conventional alkali or alkaline earth modifiers that primarily neutralize acid sites, Nb_2_O_5_ can introduce additional Lewis acidity, participate in redox cycles, and modify the accessibility of acid sites through interactions with framework hydroxyl groups. These multifunctional properties make niobium a unique modifier for tuning zeolite catalytic behavior in deoxygenation reactions. Although ZSM-5, Y zeolite, and Nb_2_O_5_-modified catalysts have each been investigated for the upgrading of biomass-derived feedstocks, direct comparisons between MFI- and FAU-type zeolites modified through identical ion-exchange and Nb_2_O_5_ impregnation procedures remain limited.^29^

Therefore, the objective of this study is to evaluate the catalytic performance of ZSM-5 and NaY zeolites modified through ammonium ion exchange and Nb_2_O_5_ impregnation in the hydrocracking of crude soybean oil, with emphasis on correlating changes in acidity, structural properties, and catalytic behavior with product distribution and deoxygenation pathways.

## 2 Materials and Methods

### 2.1 Catalyst Preparation

The catalysts were prepared using the commercial zeolites NaY and ZSM-5 (supplied by Fábrica Carioca de Catalisadores) through ion exchange with ammonium chloride followed by wet impregnation with 5 wt % niobium pentoxide.

#### 2.1.1 Ion-Exchange Procedure

The zeolites were subjected to ion exchange according to the methodology described by Zandonai et al.^30^ The replacement of sodium ions by protonic sites was performed indirectly via ammonium chloride (NH_4_Cl). The procedure was carried out in a batch reactor equipped with magnetic stirring, maintaining a solids content of 10 wt %.

Initially, the zeolite was dispersed in water and stirred at room temperature for 12 h to ensure adequate suspension and homogenization. Subsequently, an aqueous solution of NH_4_Cl was added, and the mixture was stirred for 10 min. The pH of the suspension was monitored and adjusted to a final value between 4.5 and 4.7 using 0.1 mol·L^–1^ ammonium hydroxide (NH_4_OH) and hydrochloric acid (HCl) solutions.

The suspension was then maintained at 75 °C under continuous stirring for 2 h. After completion of the exchange step, the solid was filtered and washed three times with deionized water, using a washing volume equivalent to the volume of the filter cake. The material was dried at 100 °C and subsequently calcined in a muffle furnace at a final temperature of 550 °C for 6 h to obtain the protonic form of the zeolite.

#### 2.1.2 Wet Impregnation with Niobium

Wet impregnation was performed according to the method described by Campanati et al.^31^ The zeolite (previously dried at 100 °C for 24 h) was weighed to obtain a final catalyst mass of 10 g. A total of 1.725 g of ammonium niobium oxalate (NH_4_NbO­(C_2_O_4_)_2_·H_2_O) was dissolved in 50 mL of water and added to the support to achieve a nominal loading of 5 wt % Nb_2_O_5_.

The resulting suspension was subjected to solvent removal under reduced pressure using a rotary evaporator until complete drying. The impregnated material was further dried in an oven at 70 °C for 24 h, pelletized under a pressure of 3000 kgf·cm^–2^, and finally calcined at 550 °C for 4 h.

### 2.2 Catalyst Characterization

The catalysts prepared in this study were comprehensively characterized using physicochemical techniques to determine their elemental composition, textural properties, crystalline structure, surface acidity, and morphology.

The chemical composition was determined by flame atomic absorption spectrometry (FAAS) using a SpectrAA instrument (Varian). This analysis was used to determine the bulk Si/Al ratios of the samples.

Textural properties were evaluated by nitrogen adsorption–desorption isotherms obtained using a NOVA analyzer (Quantachrome Instruments). Prior to analysis, the samples were degassed at 300 °C to remove physically adsorbed species and ensure accurate surface area and porosity measurements.

The crystalline structure was investigated by X-ray diffraction (XRD) using a D8 Advance diffractometer (Bruker). Diffractograms were recorded over a 2θ range of 5°–80°, employing CuKα radiation, a scanning rate of 0.75°·min^–1^, an operating voltage of 40 kV, and a current of 35 mA.

Fourier Transform Infrared Spectroscopy (FTIR) analyses were carried out using a Varian spectrometer in transmission mode with KBr pellets. Spectra were recorded in the 400–4000 cm^–1^ range, with a resolution of 8 cm^–1^, 100 accumulated scans, and a scanning frequency of 5 kHz. A background spectrum was collected using standard KBr under identical conditions.

Surface acidity was assessed by temperature-programmed desorption of ammonia (NH_3_-TPD), performed at the Catalysis Laboratory (DEQ/UEM) using a CHEMBET 3000 instrument (Quantachrome Instruments). This technique enabled the determination of the total acidity and the distribution of acid site strength.

Morphological characteristics were examined by scanning electron microscopy (SEM). Prior to analysis, the samples were sputter-coated with gold using an IC-50 ION COAT system (Shimadzu) to improve electrical conductivity. The micrographs were subsequently obtained using an SS-550 microscope (Shimadzu).

### 2.3 Catalytic Tests

The catalytic performance of the prepared materials was evaluated in a continuous-flow fixed-bed reactor system, as depicted in Figure S1. All experiments were conducted under identical operating conditions to ensure reproducibility and allow direct comparison among catalysts. For each catalytic run, 1.0 g of catalyst (particle size 0.8–1.0 mm) was loaded into the reactor. To improve feed distribution and prevent channeling, 0.05 g of inert silica was placed both below and above the catalyst bed, promoting better dispersion of the soybean oil throughout the catalytic layer.

Prior to reaction, the catalyst was subjected to an in situ thermal pretreatment at 450 °C under a nitrogen flow of 42 mL·min^–1^ for 1 h to remove adsorbed moisture and other volatile species from the zeolite pores. After the pretreatment step, the system was switched to hydrogen while maintaining a flow rate of 42 mL·min^–1^, and the reaction was carried out at 450 °C under atmospheric pressure. Crude soybean oil was fed into the reactor at a constant flow rate of 4 mL·h^–1^, corresponding to a weight hourly space velocity (WHSV) of approximately 3.5 h^–1^, using a piston-type electric pump. The effluent stream from the reactor was directed to a phase separator containing a water–ice mixture to promote condensation of the liquid products. Liquid and gaseous products were collected at 30 min intervals for subsequent analysis.

The catalytic experiments reported in this work were performed under identical operating conditions for comparative purposes. Replicate catalytic runs were not conducted; therefore, statistical parameters such as standard deviations and confidence intervals are not available. Consequently, the reported differences among catalysts should be interpreted as observed experimental trends rather than statistically validated differences.

### 2.4 Product Analysis

The gaseous products were analyzed by gas chromatography using an Agilent 7890A gas chromatograph equipped with HP-Plot/U and HP-Molesieve capillary columns (30 m × 0.535 mm × 20.0 μm). Compound identification was performed by injecting certified standard gas mixtures containing N_2_, H_2_, CO, CO_2_, CH_4_, C_2_H_6_, C_2_H_4_, C_3_H_8_, C_3_H_6_, and C_4_H_10_, allowing accurate peak assignment and quantification.

For liquid product analysis, samples were subjected to a derivatization step prior to chromatographic determination, following the acid-catalyzed methylation methodology proposed by Hartman and Lago.^32^ The resulting methyl esters were analyzed using a GC–MS system composed of a FOCUS GC gas chromatograph coupled to a DSQ II mass spectrometer (Thermo Electron). Compound identification was based on comparison of the obtained mass spectra with those available in the instrument’s spectral library. The relative chromatographic peak areas of the identified compounds were normalized and used to determine their relative proportions in the liquid products. The identified compounds were subsequently grouped and expressed as mass percentages according to the following categories: (i) gaseous hydrocarbons containing up to five carbon atoms (C_1_–C_5_); (ii) light hydrocarbons with six to eight carbon atoms (C_6_–C_8_); (iii) heavier hydrocarbons containing nine to 18 carbon atoms (C_9_–C_18_); (iv) oxygenated compounds corresponding to fatty acids derived from triglycerides; (v) oxygenated radicals; and (vi) CO_2_ and H_2_O.

## 3 Results and Discussion

### 3.1 Bulk Si/Al Ratio

The bulk Si/Al ratios of the catalysts were determined by atomic absorption spectrometry (Table 1). It is important to note that this technique provides an overall elemental composition of the samples and does not exclusively reflect framework (structural) atoms, as commonly assessed by solid-state NMR or other local structure-sensitive techniques.

**Table 1:** Bulk Si/Al Ratio Determined by Atomic Absorption

Catalysts	Si/Al (bulk)
ZSM-5	13.62
ZSM-5/Nb_2_O_5_	13.28
HZSM-5	11.35
HZSM-5/Nb_2_O_5_	13.95
NaY	5.11
NaY/Nb_2_O_5_	5.13
HY	3.29
HY/Nb_2_O_5_	3.43

After the ion-exchange process, variations in the bulk Si/Al ratio were observed for the zeolite-based materials. These changes are not necessarily indicative of framework dealumination or silicon leaching, as no direct experimental evidence was obtained in this study. Instead, the observed differences may reflect apparent compositional variations associated with the treatment procedure, including sample heterogeneity, experimental uncertainty, and the inherent sensitivity of the analytical method. In contrast, niobium impregnation did not significantly alter the bulk Si/Al ratio, indicating that the wet impregnation method preserved the overall framework composition.^33^

### 3.2 Acidity by NH_3_-TPD

The acidity of the catalysts was evaluated by temperature-programmed desorption of ammonia (NH_3_-TPD), and the results are summarized in Table 2. The ZSM-5-based catalysts exhibited two distinct ammonia desorption regions: a low-temperature peak (242–261 °C) associated with weak acid sites and a high-temperature peak (405–471 °C) attributed to strong acid sites. The parent ZSM-5 zeolite presented a total acidity of 1.00 mmol NH_3_·g^–1^, with a weak site reached at approximately 252 °C and a strong site at 440 °C, consistent with literature reports for ZSM-5 materials with different SiO_2_/Al_2_O_3_ ratios.^34^

**Table 2:** Acidity and Main Desorption Temperatures of Zeolite Catalysts

		Temperature of the main peaks (°C)		Acidity (mmol NH_3_/g)	
Catalysts	Total acidity (mmol NH_3_/g)	First Peak	Second Peak	Weak site	Strong site
ZSM-5	1.00	252	440	0.52	0.48
ZSM-5/Nb_2_O_5_	1.29	255	440	0.63	0.66
HZSM-5	1.59	261	471	0.75	0.84
HZSM-5/Nb_2_O_5_	1.24	251	444	0.57	0.67
NaY	0.41	227	-	0.41	-
NaY/Nb_2_O_5_	0.75	236	-	0.75	-
HY	1.40	238	366	0.53	0.87
HY/Nb_2_O_5_	0.81	248	-	0.81	-

The NH_3_-TPD results revealed significant differences in both the concentration and strength distribution of acid sites among the catalysts after ion-exchange and Nb_2_O_5_ impregnation treatments. Notably, the effect of Nb_2_O_5_ impregnation was not uniform across all catalyst types. For NaY and ZSM-5 parent zeolites, the impregnation with Nb_2_O_5_ led to an increase in total acidity (from 0.41 to 0.75 mmol NH_3_·g^–1^ for NaY and from 1.00 to 1.29 mmol NH_3_·g^–1^ for ZSM-5), suggesting the contribution of additional acid sites associated with surface niobium oxide species. In contrast, the proton-exchanged zeolites exhibited a decrease in total acidity after Nb_2_O_5_ impregnation, accompanied by a reduction in the high-temperature desorption peaks associated with strong acid sites. This opposing behavior clearly indicates that the impregnation treatment does not act through simple neutralization of acid sites. Instead, niobium species interact with framework hydroxyl groups, potentially forming new acid sites while simultaneously blocking or modifying existing strong acid sites, leading to a redistribution of acid strength rather than uniform acidity reduction. Similar behavior has been reported for Nb-modified zeolites, where NbO_*x*_ species interact with framework hydroxyl groups and induce a redistribution of acid strength rather than a uniform increase in acidity.^35–39^

Therefore, the NH_3_-TPD results indicate that the impregnation treatment modifies the acid site landscape of the zeolites, although NH_3_-TPD alone does not allow identification of the nature or origin of the individual acid sites. The observed changes should thus be interpreted as a consequence of surface modification induced by Nb_2_O_5_ impregnation (Figure 1).

**Figure 1:**
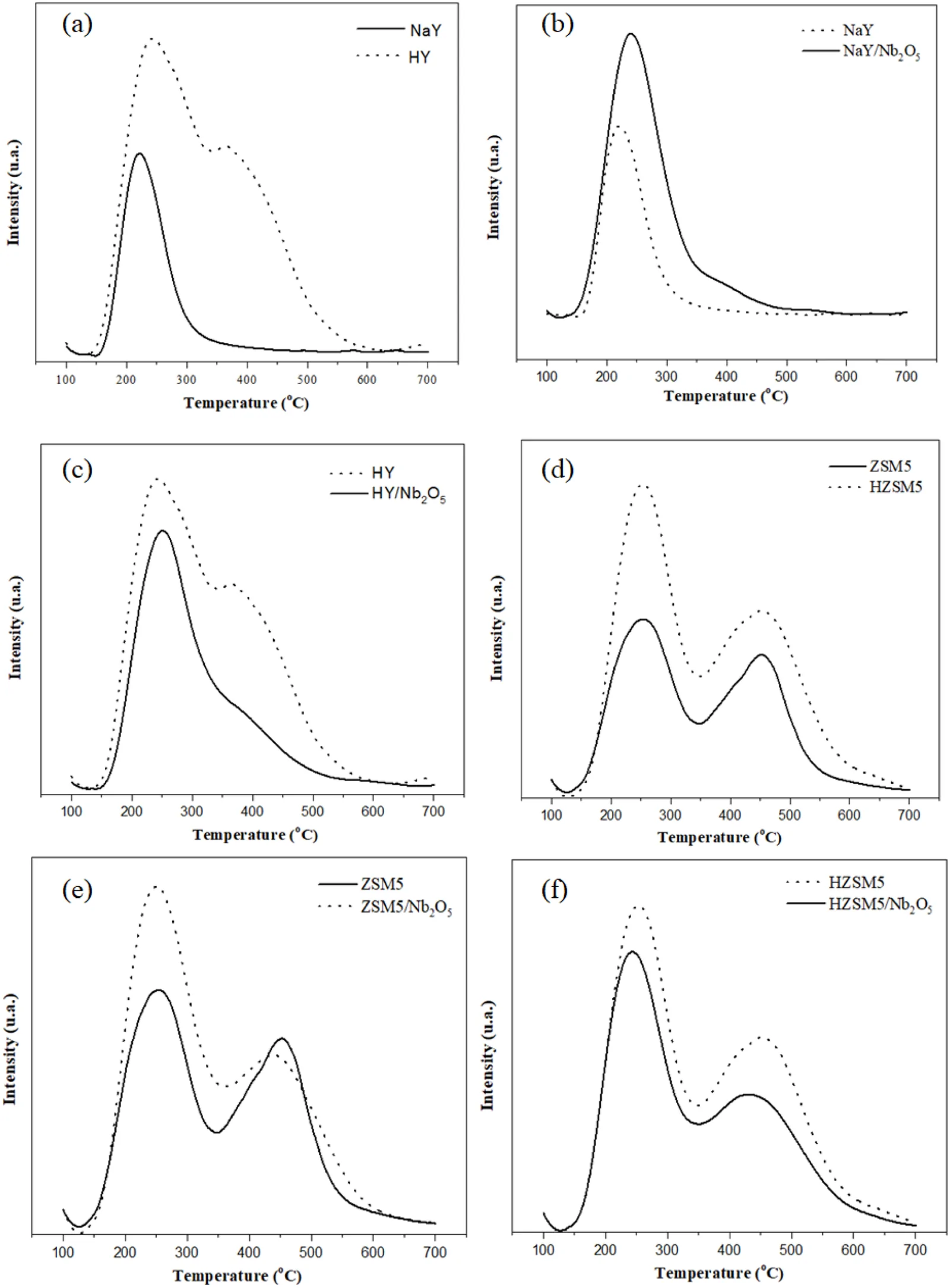
Ammonia desorption profiles: (a) NaY and HY; (b) NaY and NaY/Nb_2_O_5_; (c) HY and HY/Nb_2_O_5_; (d) ZSM-5 and HZSM-5; (e) ZSM-5 and ZSM-5/Nb_2_O_5_; (f) HZSM-5 and HZSM-5/Nb_2_O_5_.

The acidity values obtained in this work are consistent with those reported for protonic zeolites employed in biomass conversion and hydrocracking reactions. Järvinen et al.^34^ reported total acidities ranging from approximately 1.0 to 1.8 mmol NH_3_ g^–1^ for ZSM-5-based catalysts, depending on framework composition and metal modification. Similarly, the HZSM-5 catalyst investigated in the present study exhibited a total acidity of 1.59 mmol NH_3_ g^–1^, while HY presented 1.40 mmol NH_3_ g^–1^. These values confirm that the ion-exchange treatment successfully generated highly acidic materials suitable for hydrocracking applications. The increase in acidity observed after Nb_2_O_5_ incorporation into NaY and ZSM-5 is also consistent with previous reports indicating that niobium species contribute additional Lewis acidic sites and modify the overall acid strength distribution of zeolitic catalysts.^34^

### 3.3 X-ray Diffraction (XRD)

The X-ray diffractograms were analyzed using the Joint Committee on Powder Diffraction Standards (JCPDS) database for phase identification. The patterns obtained for the parent ZSM-5 and NaY zeolites confirmed their characteristic crystalline structures, consistent with typical MFI- and FAU-type frameworks.^38^

For the NaY-based materials, the ion-exchange process—where Na^+^ cations were replaced by H^+^ (via NH_4_^+^ exchange followed by calcination)—resulted in noticeable changes in the intensity of the diffraction peaks. Although the overall FAU structure was preserved, modifications in peak intensities were observed after exchange, which can be attributed to the redistribution of extra-framework cations responsible for balancing the negative charge of the aluminosilicate framework. This redistribution affects the electron density within the unit cell, leading to variations in diffraction intensity without significant structural collapse.^33,40^

No characteristic diffraction peaks corresponding to crystalline Nb_2_O_5_ phases were detected in the impregnated samples. The absence of these reflections is consistent with the presence of highly dispersed or amorphous niobium oxide species, or with crystallite sizes below the detection limit of conventional X-ray diffraction. At the nominal Nb_2_O_5_ loading employed in this study (5 wt %), strong interactions between niobium oxide species and the zeolite surface may inhibit the formation of detectable crystalline Nb_2_O_5_ domains.^41,42^

In the case of ZSM-5-based catalysts, the ion-exchange treatment did not alter the MFI framework structure, as evidenced by the preservation of the characteristic diffraction pattern (Figure 2a). Only a slight decrease in peak intensity was observed compared to the parent ZSM-5, indicating minor structural modifications and high framework stability. As observed for the Y zeolite, no distinct diffraction peaks associated with Nb_2_O_5_ were detected in the Nb-impregnated ZSM-5 samples.^43^

**Figure 2:**
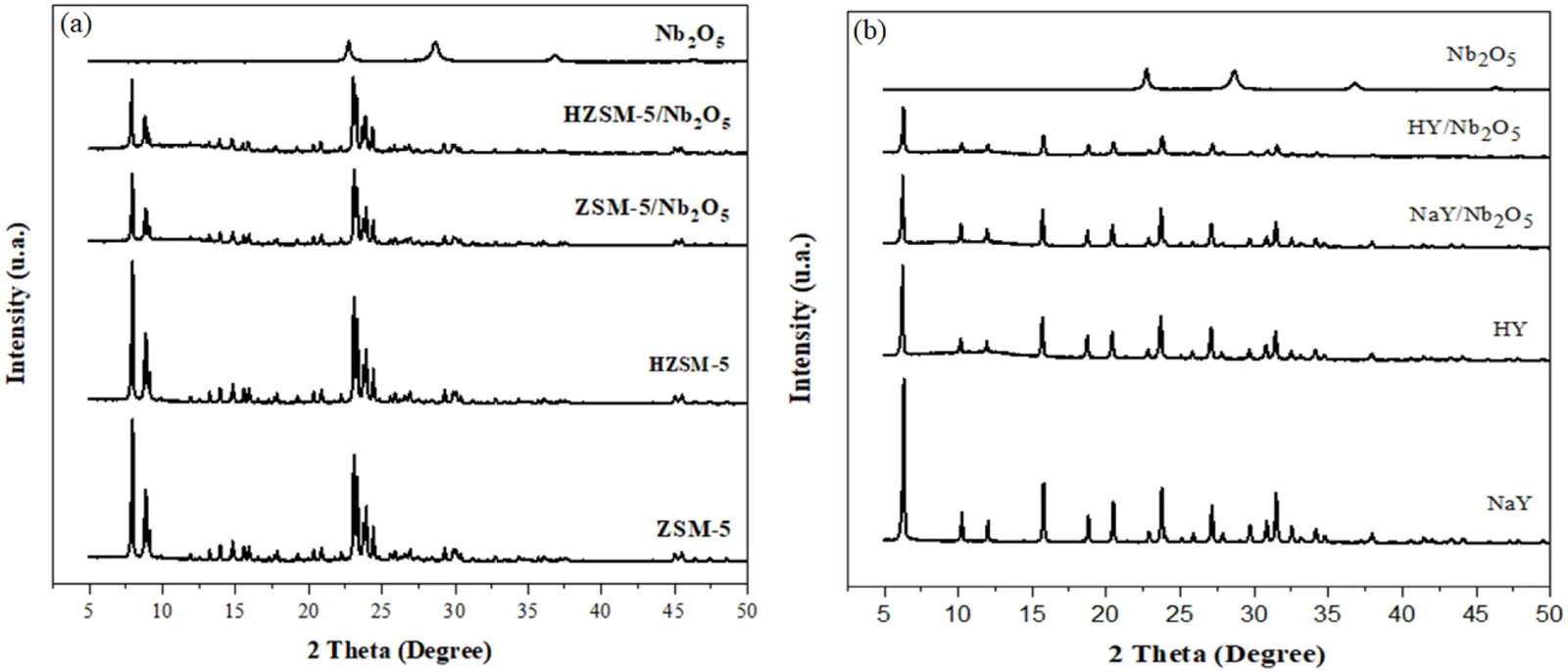
XRD patterns: (a) ZSM-5 and HZSM-5 impregnated with Nb_2_O_5_; (b) NaY and HY impregnated with Nb_2_O_5_.

The phase behavior of Nb_2_O_5_ is known to depend on both the metal loading and the degree of interaction with the support. At low surface coverage (below monolayer formation), niobium oxide remains highly dispersed and typically does not form detectable crystalline phases, even after calcination at elevated temperatures. In contrast, at higher loadings approaching or exceeding monolayer coverage, increased surface diffusion can promote crystallization of Nb_2_O_5_ at comparatively lower temperatures.^42,44^

### 3.4 Textural Properties by N_2_ Adsorption–Desorption (BET Analysis)

The nitrogen adsorption–desorption isotherms of the parent and modified zeolites are presented in Figure 3. These isotherms provide important information regarding pore structure, surface area, and the textural modifications induced by ion exchange and niobium impregnation.

**Figure 3:**
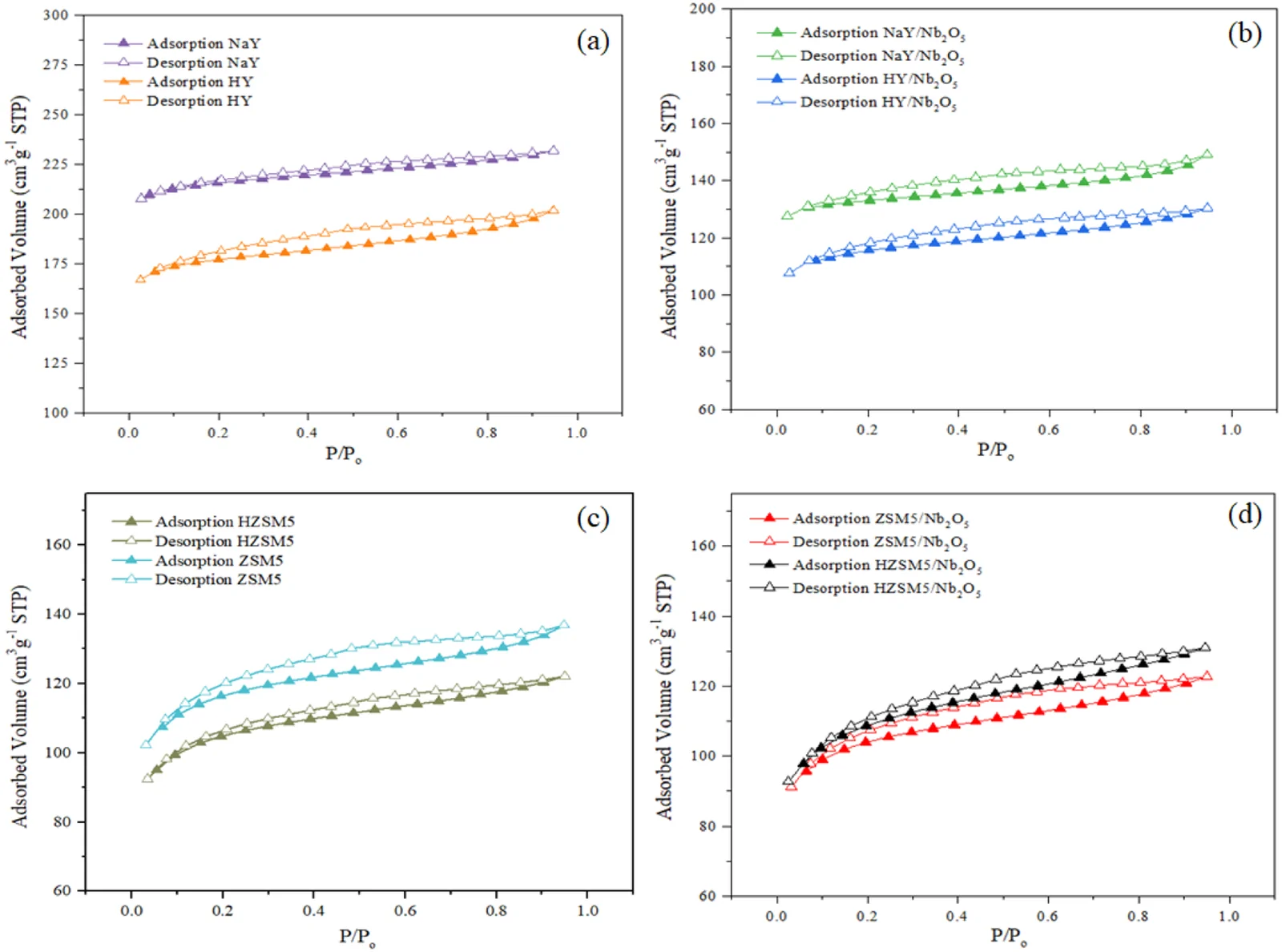
Nitrogen adsorption–desorption isotherms: (a) NaY and HY; (b) NaY/Nb_2_O_5_ and HY/Nb_2_O_5_; (c) ZSM-5 and HZSM-5; (d) ZSM-5/Nb_2_O_5_ and HZSM-5/Nb_2_O_5_.

All catalysts derived from ZSM-5 exhibited type IV isotherms with hysteresis loops, characteristic of materials containing both microporous structures and secondary mesoporosity. The presence of hysteresis suggests capillary condensation phenomena associated with intercrystalline voids or structural defects generated during treatment. After impregnation with Nb_2_O_5_ (Figure 3d), a reduction in the volume of adsorbed N_2_ was observed, indicating partial pore blocking or occupation of micropores by niobium species dispersed within the zeolite framework.

In contrast, NaY-based materials displayed type I isotherms (Figure 3a), typical of predominantly microporous solids that closely follow the Langmuir adsorption model. This behavior confirms the well-defined microporous nature of the FAU-type structure. Similar to the ZSM-5 system, niobium impregnation in NaY also led to a decrease in the adsorbed N_2_ volume (Figure 3b), suggesting partial filling or obstruction of microporous channels.

The BET surface area, micropore area, micropore volume, and average pore diameter of the catalysts are summarized in Table 3. The quantitative analysis confirms the trends observed in the isotherms, particularly the reduction in specific surface area following oxide impregnation. This behavior is consistent with the XRD results, where a decrease in peak intensity was observed, suggesting partial loss of crystallinity or pore accessibility.

**Table 3:** Textural Properties of the Catalysts

Catalysts	BET area (m^2^/g)	Micropore area (m^2^/g)	Micropore volume (cm^3^/g)	Mean pore size (Å)
ZSM-5	342.7	217.1	0.1071	10.43
HZSM-5	358.1	242.2	0.1285	10.70
ZSM-5/Nb_2_O_5_	320.8	214.9	0.1137	10.72
HZSM-5/Nb_2_O_5_	324.3	200.9	0.1128	11.69
NaY	548.2	491.2	0.3078	5.84
HY	421.6	359.7	0.2460	7.54
NaY/Nb_2_O_5_	473.2	443.8	0.2667	5.00
HY/Nb_2_O_5_	289.0	242.8	0.1578	8.60

After ion exchange, the BET surface area of HZSM-5 increased by approximately 4.5% relative to ZSM-5. This increase may be attributed to structural rearrangements during calcination, possibly involving the removal of amorphous debris and the generation of additional accessible microporous volume, as evidenced by the increase in micropore volume.

The BET surface areas obtained for ZSM-5 (342.7 m^2^·g^–1^) and HZSM-5 (358.1 m^2^·g^–1^) are consistent with values reported by Otieno et al.^45^ Similarly, the values measured for ZSM-5/Nb_2_O_5_ (320.8 m^2^·g^–1^) and HZSM-5/Nb_2_O_5_ (324.3 m^2^·g^–1^) are in agreement with those reported in the same study, confirming that niobium incorporation leads to moderate surface area reduction while preserving the overall porous structure.

Among all materials investigated, NaY exhibited the highest BET surface area (548.2 m^2^·g^–1^), confirming its highly microporous character.^46^ However, the ion-exchanged and Nb_2_O_5_-impregnated NaY-based catalysts showed a considerable decrease in surface area, with reductions of 23% for HY and 13.7% for NaY/Nb_2_O_5_ relative to the parent NaY. This reduction is consistent with the XRD results, where lower peak intensities indicated a decrease in crystallinity and possible structural alterations.

Overall, the BET analysis demonstrates that both ion exchange and niobium impregnation significantly influence the textural properties of the zeolitic materials, primarily through changes in micropore accessibility, partial pore blocking, and modifications in structural order, all of which may directly impact catalytic performance.

It should be noted that the micropore volumes reported in Table 3 were calculated using the t-plot method and therefore do not necessarily scale directly with the total adsorbed nitrogen volume observed in the adsorption–desorption isotherms. Consequently, qualitative visual comparison of the isotherms may not fully reflect the calculated microporous contribution of each catalyst.

The textural properties obtained are comparable to those reported for similar zeolitic catalysts in the literature. ZSM-5 catalysts typically exhibit BET surface areas between 300 and 400 m^2^ g^–1^, whereas NaY materials generally present values above 500 m^2^ g^–1^. The results obtained in the present study fall within these ranges, confirming the preservation of the zeolite frameworks after modification. The moderate reduction in surface area after Nb_2_O_5_ impregnation is also consistent with previous studies, where niobium species partially occupied pore entrances or external surface regions without causing severe structural collapse.^45,46^

### 3.5 Fourier Transform Infrared Spectroscopy (FTIR)

The FTIR spectra of the pure ZSM-5 zeolite (Figure 4) exhibited characteristic vibrational bands consistent with the MFI framework and previously reported assignments. In the low-wavenumber region (650–500 cm^–1^), bands associated with the double-ring vibrations of the zeolite framework were observed, in agreement with literature assignments for ZSM-5 structures. The band near 450 cm^–1^ is attributable to angular vibrations of Al–O–Si linkages, while the region between 790 and 800 cm^–1^ corresponds to external symmetric stretching modes of the T–O–T (T = Si or Al) network. A band at approximately 1229 cm^–1^ can be assigned to external asymmetric stretching vibrations of Al–O–Si/Si–O–Al linkages, in line with previous FTIR characterizations of ZSM-5 zeolites.^47,48^

**Figure 4:**
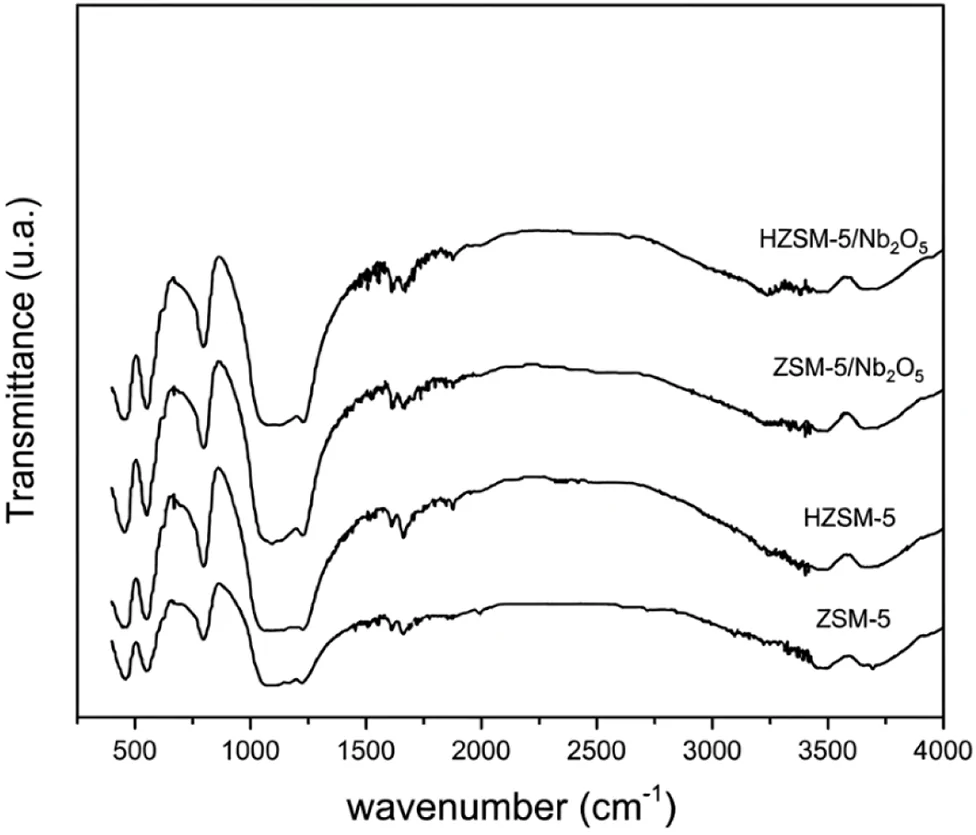
FTIR spectrum for ZSM-5 catalysts.

In the mid-infrared region between 1500 and 1700 cm^–1^, broad absorptions are attributed to molecular water adsorbed on the catalyst surface, likely arising from residual moisture in the sample or from the KBr matrix used for pellet preparation.^49^

The spectral region between 600 and 950 cm^–1^, where Nb–O stretching modes are typically expected for crystalline niobium oxides (600–900 cm^–1^), did not exhibit distinct features attributable to Nb_2_O_5_ species. This absence supports the hypothesis that niobium pentoxide was not incorporated into the zeolite framework but is either highly dispersed on the external surface or partially confined within the zeolite cavities, forming noncrystalline or highly dispersed surface species that are not resolved in the FTIR spectrum.^50,51^

The FTIR spectra of the NaY catalysts are presented in Figure 5. Broad bands centered around 3460 cm^–1^ and 1640 cm^–1^ are assigned to adsorbed water and surface hydroxyl groups, including bridging Si–OH and extraframework Al–OH species, consistent with FTIR studies of Y-type zeolites. The intense band at approximately 1150 cm^–1^ corresponds to asymmetric T–O–T stretching vibrations (T = Si or Al) characteristic of the faujasite framework. Bands near 800 cm^–1^ and 460 cm^–1^ are attributed to symmetric stretching and bending (flex) modes of the T–O–T network, respectively, which are well documented for FAU structures.^52,53^

**Figure 5:**
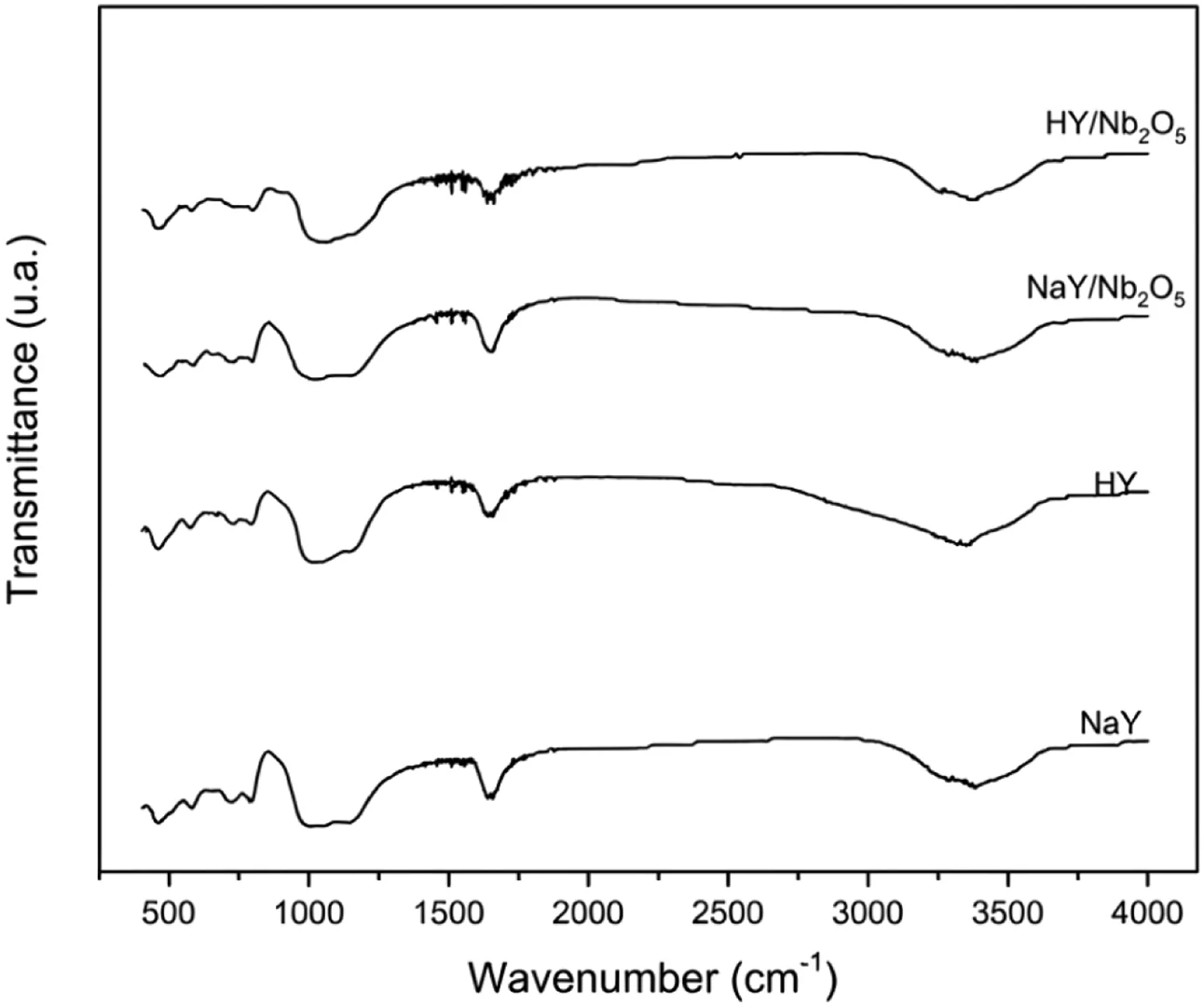
FTIR spectrum for NaY catalysts.

The band at 3460 cm^–1^ is specifically associated with Si–OH groups on the external surface of the NaY zeolite, an assignment consistent with previous structural FTIR analyses of hydrated Y zeolites. Between 400 and 1000 cm^–1^, a general reduction in band intensity was observed after ion exchange and impregnation, suggesting changes in the local environment of the framework vibrations and in the concentration of hydroxyl groups. This trend is consistent with the textural and structural modifications inferred from XRD and adsorption analyses.^54,55^

### 3.6 Scanning Electron Microscopy (SEM) Analysis

The scanning electron microscopy (SEM) analysis revealed that the ZSM-5 and HZSM-5 zeolites exhibit orthorhombic morphology, with crystallites predominantly arranged in a prismatic form (Figure 6). Comparison of the ZSM-5 micrographs with those of its proton-exchanged form (HZSM-5) showed no significant changes in crystallite size or surface morphology, indicating that the protonation process does not substantially alter the external surface of the ZSM-5 framework.^56,57^

**Figure 6:**
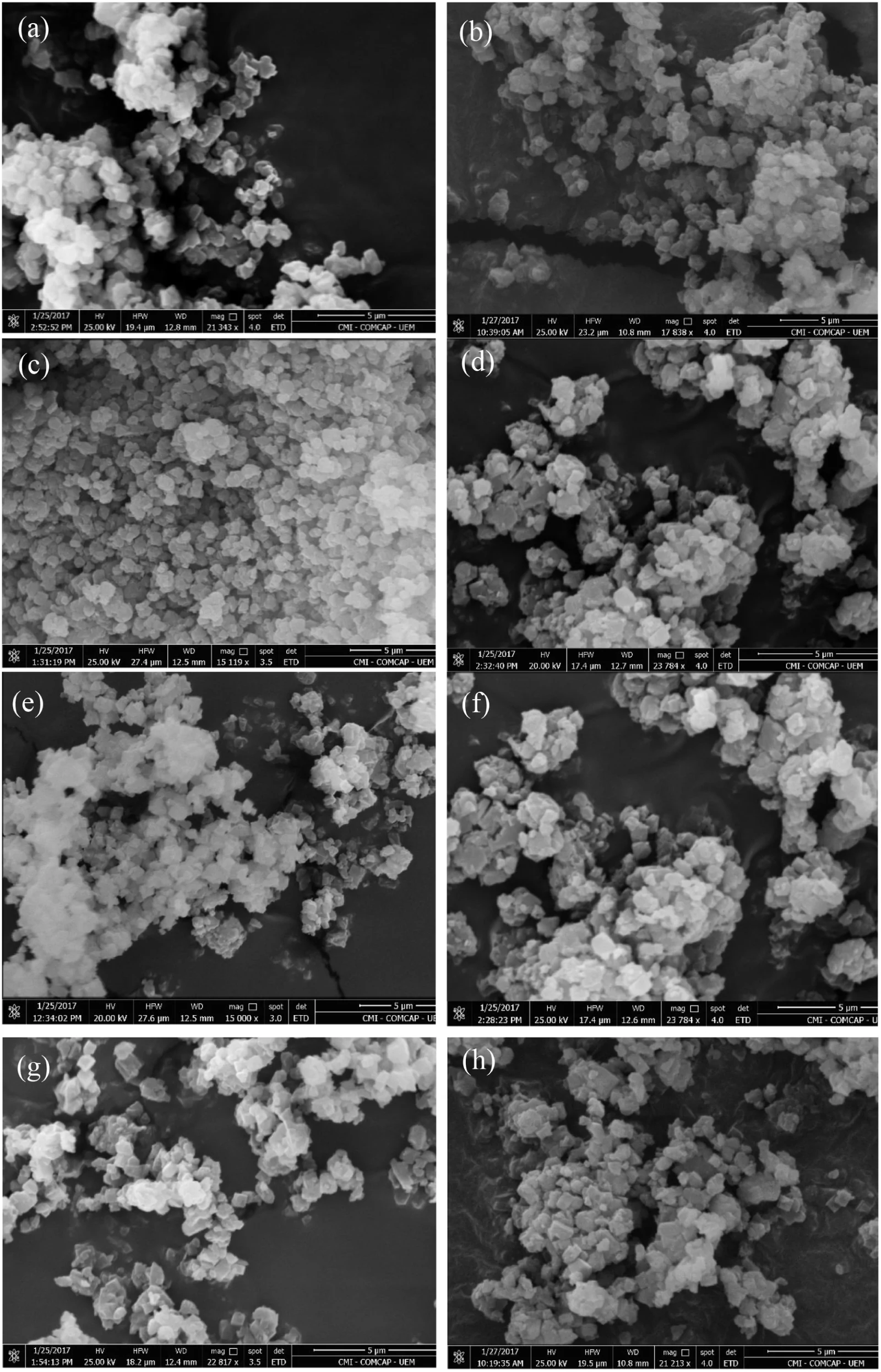
SEM micrographs of catalysts: (a) HY, (b) HY/Nb_2_O_5_, (c) NaY, (d) NaY/Nb_2_O_5_, (e) ZSM-5, (f) ZSM-5/Nb_2_O_5_, (g) HZSM-5, (h) HZSM-5/Nb_2_O_5_.

The niobium-impregnated ZSM-5 samples displayed a small fraction of amorphous material, suggesting a slight reduction in crystallite size, consistent with the observations from X-ray diffraction (XRD) analysis. Particle agglomeration observed in all ZSM-5-based catalysts can likely be attributed to the thermal treatments applied during catalyst preparation.^58,59^

SEM images of NaY zeolite samples subjected to ion exchange and Nb_2_O_5_ impregnation are presented in Figure 6. Pure NaY exhibits a cubic morphology, and the ion-exchange process with ammonia does not significantly alter the crystal size or morphology, in agreement with prior studies. Although it has been reported that higher Nb_2_O_5_ loadings can lead to increased formation of amorphous material, the Nb_2_O_5_ content used in this work was insufficient to cause substantial crystallinity loss. These results align with literature observations that HY-type zeolites are more susceptible to structural degradation than ZSM-5 under similar treatments and that the effect depends strongly on the zeolite framework type.^60,61^

### 3.7 Product Distribution over ZSM-5-Based Catalyst

The product distribution obtained over the ZSM-5 zeolite-based catalysts at 30 min of reaction is presented in Figure 7, whereas the corresponding results at 60 min are shown in Figure 8. As observed, gaseous hydrocarbons constitute the majority of the products formed, and minor amounts of light hydrocarbons were also detected, indicating that the reaction temperature of 450 °C is sufficient to cleave the long-chain organic molecules.

**Figure 7:**
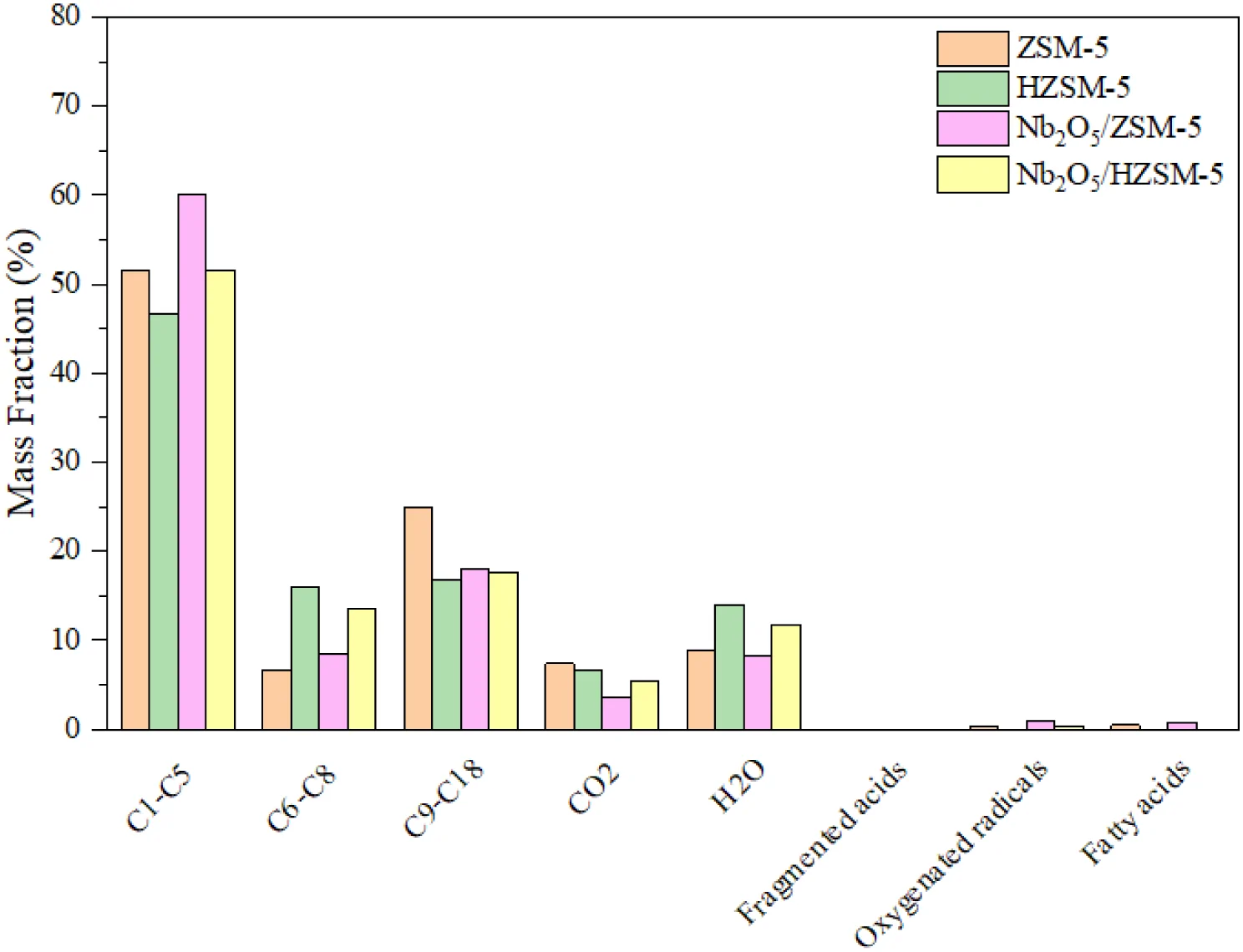
Product distribution obtained during crude soybean oil hydrocracking over ZSM-5-based catalysts after 30 min of reaction at 450 °C under hydrogen flow. Products were grouped into gaseous hydrocarbons (C_1_–C_5_), light liquid hydrocarbons (C_6_–C_8_), heavy hydrocarbons (C_9_–C_18_), oxygenated compounds, CO_2_, and H_2_O.

**Figure 8:**
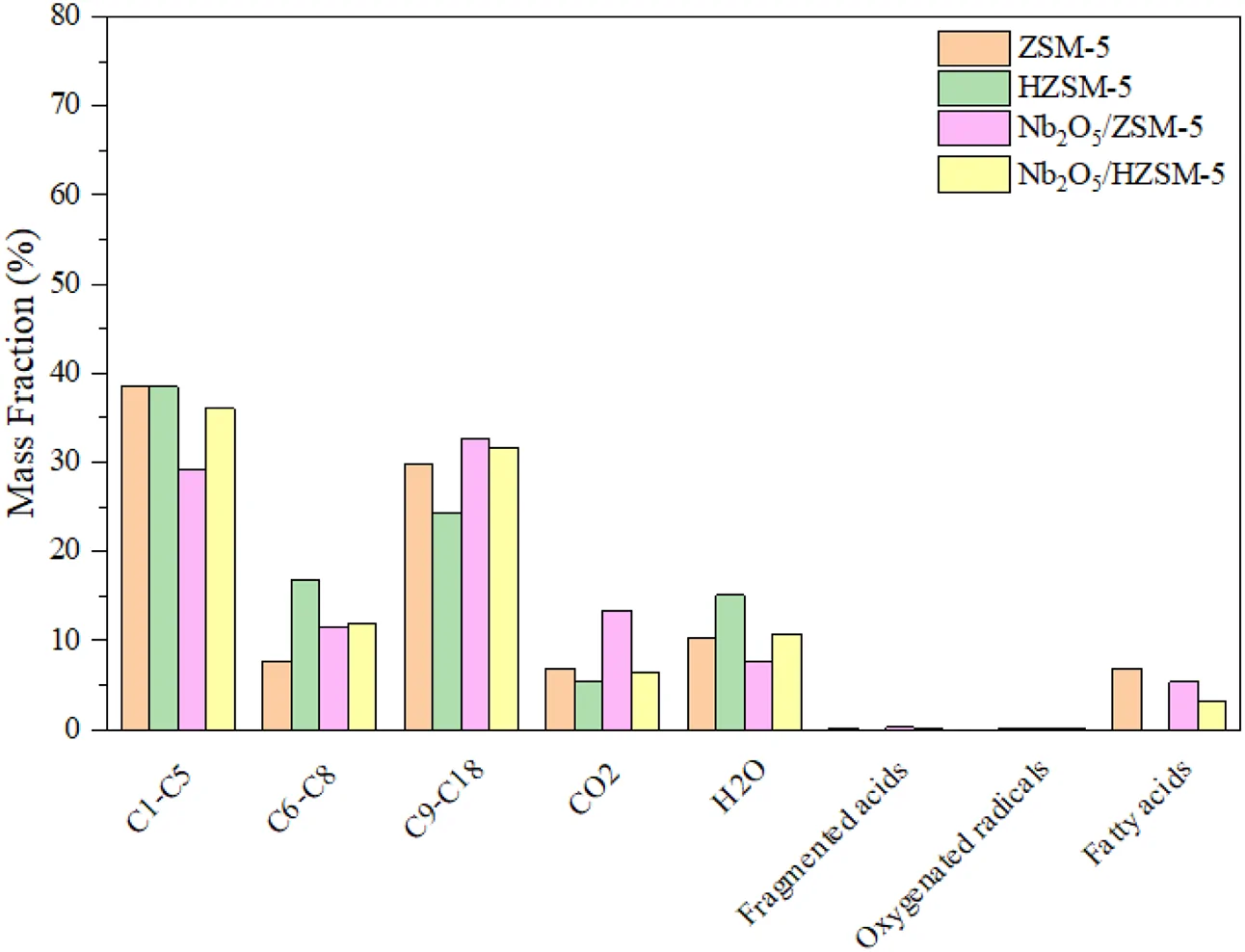
Product distribution in the hydrocracking of soybean oil over ZSM-5-based catalysts after 60 min of reaction.

During the 30 min reaction period, both catalysts generated more water than carbon dioxide, suggesting a greater contribution of the deoxygenation pathway via hydrogenation of fatty acids. Decarboxylation also occurred; however, ion exchange decreased this pathway, resulting in higher water formation relative to CO_2_ for HZSM-5. The CO_2_ concentration decreased by ∼6% over 60 min, suggesting a lower relative contribution of decarboxylation to the deoxygenation using ZSM-5. The observed increase in organic acids after 60 min using ZSM-5 reflects changes in product distribution with reaction time, reducing molecular fragmentation.^62–65^

Ion exchange significantly enhanced the total acidity of ZSM-5. Consequently, HZSM-5 displayed increased selectivity during the first 30 min, with products largely free of oxygenates. Over 60 min, HZSM-5 selectivity remained higher than that of ZSM-5, demonstrating the effectiveness of protonation in enhancing hydrocarbon selectivity. Nevertheless, a slight decline in selectivity over time was observed. Although this behavior may be associated with changes occurring during the reaction, including possible modifications in the accessibility of active sites, the present data do not allow a conclusive assessment of catalyst deactivation because no postreaction catalyst characterization was performed.^66,67^

Comparing zeolites before and after ion exchange, the C1–C5 fraction remained nearly unchanged over the last 30 min, the C9–C18 fraction decreased by ∼18%, and the C6–C8 fraction increased by ∼120%, indicating that protonation continues to favor light hydrocarbons during the later reaction stage. HZSM-5 showed an increase in heavy hydrocarbons, which may be associated with aromatic formation via dehydrogenation at strong acidic sites. Overall, HZSM-5 in its protonated form was more effective in soybean oil hydrocracking than the parent ZSM-5 due to enhanced deoxygenation, favoring biofuel production.^30,68^

Niobium pentoxide incorporation altered the crystallinity and acidity of ZSM-5 by ∼29%. During the first 30 min, C1–C5 and C6–C8 production increased by 18.96% and 28.25%, respectively, indicating that Nb_2_O_5_ promotes gasoline-range biofuel formation. The C9–C18 fraction decreased with increasing acidity, which may be associated with a greater contribution of aromatization and dehydrogenation reactions.^51^

Over the two reaction intervals, the light and heavy liquid product distribution for ZSM-5/Nb_2_O_5_ decreased in the latter 30 min, indicating progressive fragmentation of long-chain components and increased heavy liquid formation due to excessive dehydrogenation. A similar inversion of H_2_O/CO_2_ ratios was observed, suggesting a change in the relative contributions of hydrogenation and decarboxylation pathways between the two reaction stages. The formation of oxygenated compounds, including fatty acids, during the later reaction stage indicates a reduction in deoxygenation efficiency and molecular fragmentation. However, the underlying causes cannot be conclusively established from the available data because spent catalyst characterization was not performed.^64,69^

Compared to pure HZSM-5, both catalysts exhibited similar trends for gaseous and light liquid products. However, heavy liquid hydrocarbons increased by 4.58%, suggesting enhanced aromatization and boiling-point elevation. After 60 min, heavy liquid products increased further by 30.47%, which may be associated with dehydrogenation and aromatic formation.^70^

Although differences among catalysts were observed after 30 min of reaction, the product distributions became progressively more similar after 60 min. This behavior suggests that the influence of catalyst composition was more pronounced during the initial reaction stage. Because no postreaction catalyst characterization was performed, the convergence of product distributions cannot be conclusively attributed to catalyst deactivation, coke deposition, acidity loss, or structural changes. Therefore, the results should be interpreted primarily as evidence of the influence of catalyst acidity and framework characteristics on the initial stages of soybean oil conversion.

### 3.8 Product Distribution over NaY-Based Catalyst

The weak acidity of the NaY zeolite significantly influenced the fragmentation of long-chain molecules during hydrocracking. After 30 min of reaction, the liquid hydrocarbons (Figure 9) were deoxygenated but largely uncracked, indicating that the zeolite’s low acid strength limited the cleavage of carbon–carbon bonds. In the subsequent reaction stage, the production of light liquid hydrocarbons increased by approximately 200%, reflecting enhanced cracking of long-chain molecules and the generation of more components within the gasoline range. The observed predominance of H_2_O over CO_2_ suggests a greater contribution of hydrodeoxygenation via hydrogenation/dehydration, while the accumulation of fragmented organic acids indicates incomplete deoxygenation due to insufficient acid site activity.^71^

**Figure 9:**
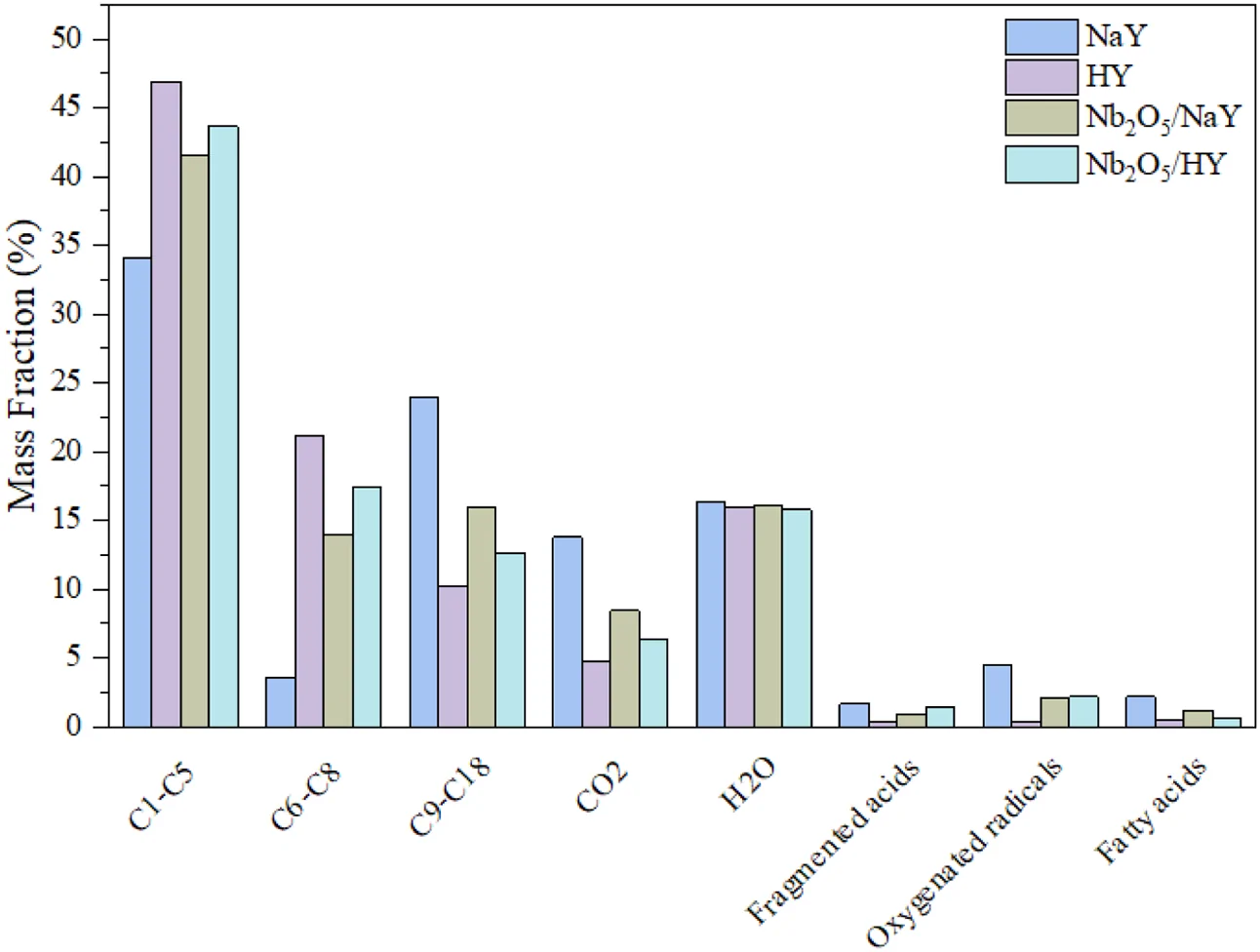
Product distribution in the hydrocracking of soybean oil over NaY-based catalysts after 30 min of reaction.

Following the ammonium ion-exchange procedure, the acidity of NaY increased from 0.41 to 1.40 mmol NH_3_·g^–1^, which altered the product distribution. The relative inversion of gas and heavy liquid products over time may be associated with changes in catalyst performance during the reaction. However, without characterization of the spent catalysts, possible causes such as coke deposition, pore blockage, acidity loss, or structural modifications cannot be distinguished. Niobium pentoxide impregnation further increased total acidity by 83%, enhancing the production of gaseous and light liquid fractions while reducing the heavy liquid fraction.^51,72^

During the final 30 min (Figure 10), NaY/Nb_2_O_5_ produced fewer gaseous hydrocarbons, whereas light and heavy liquid fractions increased due to initial changes in product distribution with reaction time, which limited chain cleavage. Consequently, deoxygenation efficiency declined, as evidenced by minor changes in CO_2_ production, a reduction in H_2_O, and a notable increase in residual acids. For HY/Nb_2_O_5_, a substantial accumulation of fragmented acids was observed after 60 min, reflecting insufficient deoxygenation despite ongoing molecular cleavage. The CO_2_ levels declined while H_2_O increased, a greater relative contribution of hydrogenation/dehydration to the deoxygenation pathway. Additionally, a reduction in gaseous product and an increase in heavy liquids were observed, possibly reflecting changes in the accessibility of active sites during the reaction. Nevertheless, the present results do not provide direct evidence for pore blockage, coke deposition, or acidity loss, and therefore these mechanisms remain speculative.

**Figure 10:**
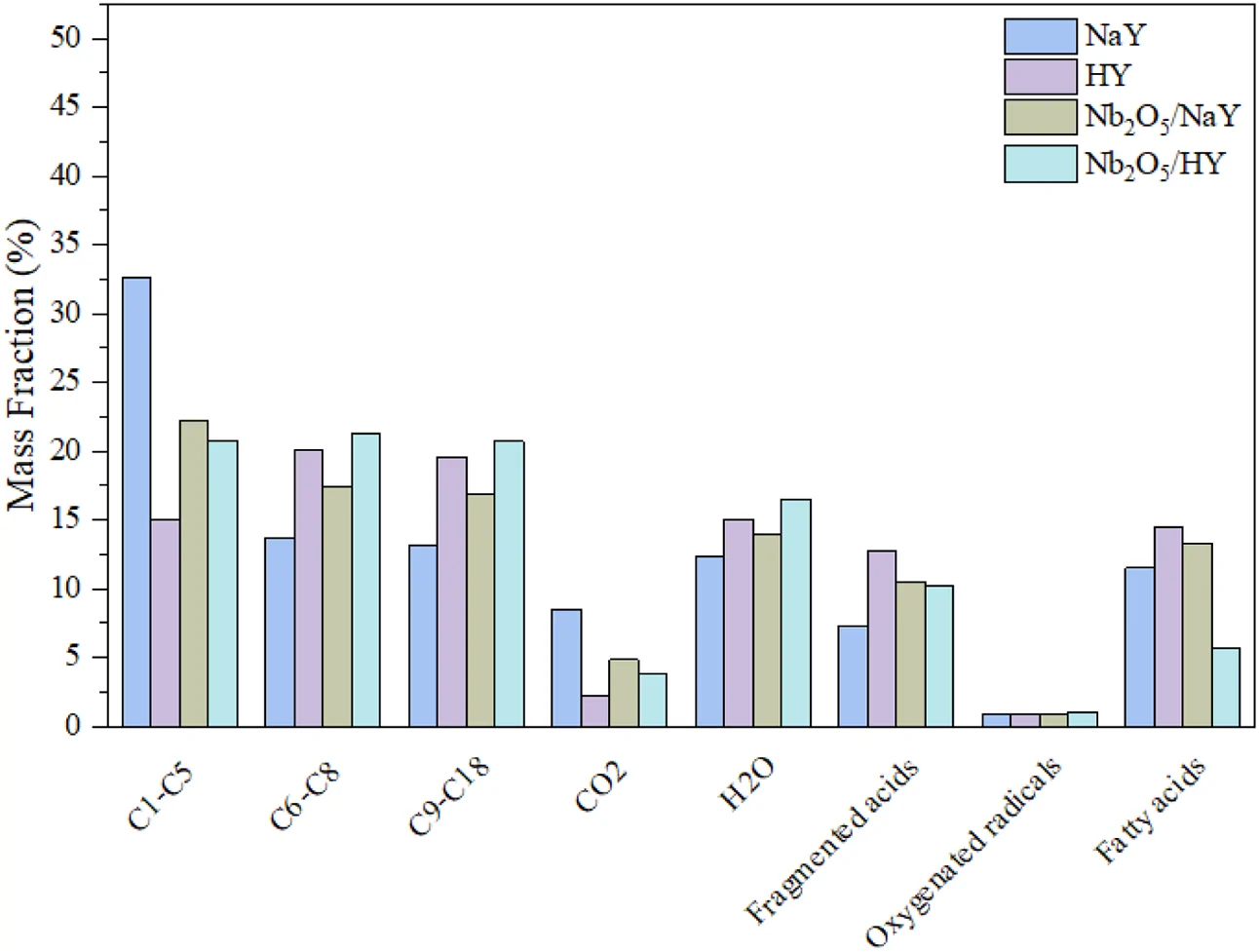
Product distribution in the hydrocracking of soybean oil over NaY-based catalysts after 60 min of reaction.

In general, ZSM-5-based catalysts generated a higher fraction of gaseous products than NaY-based catalysts, likely due to the smaller pore size facilitating deeper cracking, while heavy liquids were more prevalent over ZSM-5 due to enhanced aromatization. Conversely, NaY-based catalysts favored light liquid hydrocarbons, reflecting the predominance of weaker acid sites and limited molecular fragmentation. Across 60 min, all catalysts produced an increased gaseous fraction compared to the first 30 min, with ZSM-5 retaining higher activity in this range. The ion-exchange processes in HY and HZSM-5 enhanced light liquid hydrocarbon production in both reaction stages and reduced the heavy liquid fraction during the latter stage of hydrocracking.^72^

The catalytic behavior observed in this study is generally consistent with previous reports on vegetable oil hydrocracking over zeolitic catalysts. HZSM-5 has been widely reported to favor the production of lighter hydrocarbons due to its stronger acidity and shape-selective pore system, whereas Y-type zeolites typically produce larger fractions of liquid hydrocarbons, which may be associated with their larger pore openings and greater accessibility to bulky molecules. A similar trend has also been observed in recent studies using NaY-based catalysts for vegetable oil conversion.^73^ The present results follow the same trend, with ZSM-5-based catalysts exhibiting greater production of gaseous hydrocarbons and NaY-based catalysts favoring liquid fractions. Furthermore, the influence of niobium addition on hydrocarbon distribution agrees with literature reports describing enhanced deoxygenation and aromatization activity for Nb-modified zeolites. The combined use of proton exchange and niobium impregnation therefore represents a viable approach for tuning catalyst acidity and controlling product selectivity during soybean oil hydrocracking.^70–72^

## 4 Conclusions

ZSM-5 and NaY zeolites were successfully modified through ion exchange and Nb2O5 impregnation, producing catalysts with distinct structural and acidic properties. Importantly, the effect of Nb_2_O_5_ impregnation on acidity was not uniform: acidity increased for Na^+^-form zeolites but decreased for H^+^-form zeolites, accompanied by changes in acid strength distribution. This behavior indicates that niobium species do not simply neutralize acid sites but rather redistribute acid strength through interactions with framework hydroxyl groups. Characterization by XRD, FTIR, BET, NH_3_-TPD, and SEM confirmed that the modifications altered pore characteristics and acid site distributions while preserving the overall zeolite framework.

Hydrocracking of crude soybean oil produced hydrocarbon-rich liquid products whose compositions depended on catalyst type and reaction time. H-ZSM-5 favored the formation of paraffinic hydrocarbons, whereas H-NaY generated higher proportions of aromatic compounds. The Nb-containing catalysts exhibited different product distributions, indicating that niobium incorporation influenced catalytic behavior.

The behavior of Nb_2_O_5_-modified zeolites suggests that the combination of acidic and Nb-containing sites may contribute to cracking and dehydrogenation reactions, representing a potential advantage over simple acid neutralization achieved with alkali or alkaline earth metals. The differences observed among catalysts are consistent with the variations in acidity and textural properties identified during characterization. Although the results demonstrate that catalyst modification affects product composition, additional studies, including conversion measurements, hydrocarbon yields, carbon balances, and catalyst stability assessments, are necessary to establish catalytic selectivity and deoxygenation performance quantitatively.

## Supplementary Material



## Data Availability

All data generated or analyzed during this study are included in this published article.

## Notes

Experimental scheme of the hydrocracking module (Figure S1) (PDF)

## ◆

Department of Chemical Engineering, Western Paraná State University, R. Universitária 1619, CEP 85819-110 Cascavel, PR, Brazil
