# Supplementary material for: Tailoring Acidity and Structure of ZSM‑5 and NaY Zeolites via Ion Exchange and Nb2O5 Impregnation for Enhanced Hydrocracking of Crude Soybean Oil
Source: ACS Omega. 2026 Oct 9;11(41):64161–74. doi: 10.1021/acsomega.6c08841 (PMC13689270; doi:10.1021/acsomega.6c08841)
Supplement: Supplementary file 1 [file ao6c08841_si_001.pdf]

Tailoring Acidity and Structure of ZSM-5 and NaY Zeolites via Ion Exchange and Nb2O5 Impregnation for Enhanced Hydrocracking of Crude Soybean Oil

Taysa S. Braniz[a], [c], *Ana C. R. Aranha*[b], *Thaisa F. Coslop*[a*], *Marcos Souza*[b], *Mara H. N. O. Scaliante*[b]

[a]Postgraduate Program in Chemical Engineering, Department of Chemical Engineering, State University of Maringá, Av. Colombo 5790, Bloco D90, CEP 87020-900 Maringá, PR, Brazil

[b]Department of Chemical Engineering, State University of Maringá, Av. Colombo 5790, Bloco D90, CEP 87020-900 Maringá, PR, Brazil

[c]Present address: Department of Chemical Engineering, Western Paraná State University, R. Universitária 1619, CEP 85819-110 Cascavel, PR, Brazil

*Corresponding author: Thaisa F. Coslop

E-mail address: pg55028@uem.br

Supporting Information

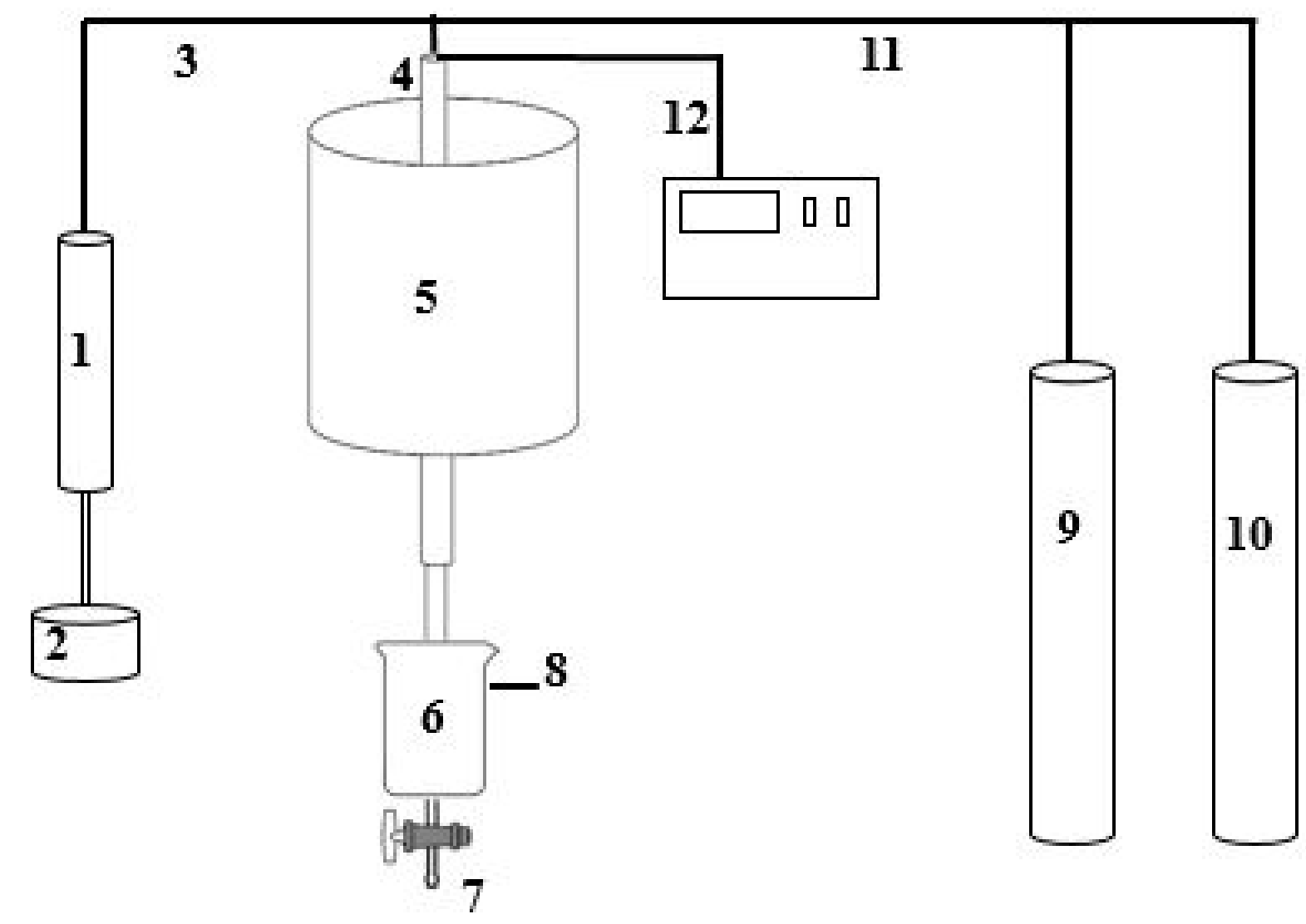

Figure S1. - Experimental scheme of the hydrocracking module.

Where: 1 – Oil reservoir; 2 – Piston-type electric pump; 3 – Oil line; 4 – Reactor; 5 – Oven; 6 – Phase separator; 7 – Liquid phase collector; 8 – Gaseous phase collector; 9 – Nitrogenic gas; 10 – Hydrogen gas; 11 – Gas line; 12 – Thermocouple.
